# 3D printed non-uniform anthropomorphic phantoms for quantitative SPECT

**DOI:** 10.1186/s40658-024-00613-7

**Published:** 2024-01-22

**Authors:** Lovisa Jessen, Johan Gustafsson, Michael Ljungberg, Selma Curkic-Kapidzic, Muris Imsirovic, Katarina Sjögreen-Gleisner

**Affiliations:** 1 Medical Radiation Physics, Lund, Lund University, Lund, Sweden; 2 Radiation Physics, Department of Hematology, Oncology and Radiation Physics, Skåne University Hospital, Lund, Sweden; 3 Skane University Hospital, Lund, Sweden

**Keywords:** Quantitative SPECT, Phantom, 3D printing, Monte Carlo, Kidneys, Thyroid

## Abstract

**Background:**

A 3D printing grid-based method was developed to construct anthropomorphic phantoms with non-uniform activity distributions, to be used for evaluation of quantitative SPECT images. The aims were to characterize the grid-based method and to evaluate its capability to provide realistically shaped phantoms with non-uniform activity distributions.

**Methods:**

Characterization of the grid structures was performed by printing grid-filled spheres. Evaluation was performed by micro-CT imaging to investigate the printing accuracy and by studying the modulation contrast ($C_{{\text{M}}}$) in SPECT images for ^177^Lu and ^99m^Tc as a function of the grid fillable-volume fraction (*FVF*) determined from weighing. The grid-based technique was applied for the construction of two kidney phantoms and two thyroid phantoms, designed using templates from the XCAT digital phantoms. The kidneys were constructed with a hollow outer container shaped as cortex, an inner grid-based structure representing medulla and a solid section representing pelvis. The thyroids consisted of two lobes printed as grid-based structures, with void hot spots within the lobes. The phantoms were filled with solutions of ^177^Lu (kidneys) or ^99m^Tc (thyroids) and imaged with SPECT. For verification, Monte Carlo simulations of SPECT imaging were performed for activity distributions corresponding to those of the printed phantoms. Measured and simulated SPECT images were compared qualitatively and quantitatively.

**Results:**

Micro-CT images showed that printing inaccuracies were mainly uniform across the grid. The relationships between the *FVF* from weighing and $C_{{\text{M}}}$ were found to be linear (*r* = 0.9995 and *r* = 0.9993 for ^177^Lu and ^99m^Tc, respectively). The *FVF*-deviations from the design were up to 15% for thyroids and 4% for kidneys, mainly related to possibilities of cleaning after printing. Measured and simulated SPECT images of kidneys and thyroids exhibited similar activity distributions and quantitative comparisons agreed well, thus verifying the grid-based method.

**Conclusions:**

We find the grid-based technique useful for the provision of 3D printed, realistically shaped, phantoms with non-uniform activity distributions, which can be used for evaluation of different quantitative methods in SPECT imaging.

**Supplementary Information:**

The online version contains supplementary material available at 10.1186/s40658-024-00613-7.

## Background

Physical phantoms are regularly used to calibrate, validate and evaluate the performance of imaging systems, image processing methods and quantification accuracy. For nuclear medicine applications, phantoms must either be radioactive themselves or enable filling with a radioactive solution without leakage. Additional desired properties are that phantoms are reusable, and can mimic clinically relevant geometries and activity distributions, preferably with thin or no non-radioactive walls between internal structures.

Commercially available phantoms range from those of stylized shapes, such as the cylindrical Jaszczak phantom or NEMA body phantoms (DataSpectrum Inc.), to those mimicking anthropomorphic shapes. While providing some of the desired properties, these phantoms are expensive and often lack in flexibility. In-house manufacturing of printed cheap and task-tailored phantoms started with the use of inkjet printers and the integration of a radioactive substance into the printing material. Layers were printed and stacked to result in a three-dimensional radioactive phantom [1]. With the introduction of 3D printing, new possibilities have emerged for manufacturing phantoms with diverse properties, using different techniques [2]. Incorporation of radioactive liquids into the printing resin was demonstrated for both ^18^F and ^99m^Tc for stereolithography (SLA) printing of spheres and cylinders [3–5]. Other printing techniques, such as fused deposition modelling (FDM), have been used for construction of phantoms in shapes of kidney, spleen and pancreas, designed from MRI or CT images or based on ICRP 110 adult computational phantoms [6–8].

Due to the poor spatial resolution of clinical SPECT and PET images, combined with effects of noise and nonlinearities intrinsic in modern SPECT reconstruction algorithms, non-uniform activity distributions may affect the results of quantitative image analysis and interpretation and thus needs to be considered in the evaluation of quantitative methods [9, 10]. The manufacturing of non-uniform phantoms was addressed by Pfaehler et al. [11] who 3D printed tumour-like structures as separate sub-compartments to be filled with different activity concentrations. A similar method involving filling of separate internal compartments was also developed for kidneys [12]. For PET and SPECT imaging, the limited spatial resolution can be exploited to construct objects that, when imaged, appear to have a variable activity concentration. By use of grids, i.e., sections with alternating solid material and voids, with dimensions and spacing that are considerably smaller than the spatial resolution, the image signal can be modulated. This technique is analogous to those used for the determination of the modulation contrast and modulation transfer function in radiography [13]. Thus, using grids of variable spacing, objects which in the images appear to have a non-uniform activity distribution can be created [14, 15]. This grid-based method avoids the cumbersome steps of using several radioactive solutions with different activity concentrations and instead enables filling of the phantom with one single solution. Another advantage is that the cold walls between phantom sub-compartments are avoided. In this study, we aimed to explore the grid-based technique for the evaluation of quantitative SPECT [15], extending it to both kidneys and thyroids, and perform detailed verification towards Monte Carlo simulated images.

Quantitative SPECT is a central tool for dosimetry in radionuclide therapy [16–18]. Examples of quantification tasks that rely on SPECT image analysis are activity estimation for kidneys in ^177^Lu peptide receptor radionuclide therapy (PRRT) and mass determination of the thyroid using ^99m^Tc-pertechnetate imaging for pre-therapeutic dosimetry in Na[^131^I]I treatment of benign thyroid disease. For both applications, patient images may exhibit non-uniform activity uptakes within the imaged regions. For kidneys in ^177^Lu PRRT, the uptake is known to vary, and after the first few hours after administration, the activity concentration in the renal pelvis is low while it is gradually higher towards the outer medulla and cortex [19, 20]. Likewise, the uptake of ^99m^Tc-pertechnetate may vary within the thyroid and exhibit regions with increased activity accumulation [21]. Methods involved in analysis of SPECT images include organ segmentation and management of partial volume effects. Most often, the strategies employed are established based on phantom imaging, using stylized phantom geometries, and tomographic reconstruction in the same manner as for patient images [22]. Evaluation of the quantitative performance and dosimetric accuracy for real patient images is challenging, since uncertainties are introduced by factors associated with the organ geometry and non-uniform activity uptakes. In order to enable experimental evaluation in geometries that are representative of patients, there is a need of phantoms that mimic patient anatomies and support the inclusion of non-uniform activity distributions.

The aim of this study was to construct anthropomorphic phantoms representing non-uniform activity distributions in kidneys during ^177^Lu PRRT and in thyroids imaged with ^99m^Tc-pertechnetate for pre-therapeutic dosimetry in Na[^131^I]I treatment for benign thyroid disease. Specifically, the aims included the production of watertight, reusable phantoms that enabled filling with a single radioactive solution and detailed verification of the produced phantoms, using micro-CT imaging, weighing and Monte Carlo simulations.

## Material and methods

Four differently shaped geometries were 3D printed: i) cubes, ii) spheres, iii) two non-uniform kidney replicas and iv) two thyroid-shaped objects with hot spots. All objects were prepared in PreForm (Formlabs Inc.) and printed with an SLA Formlabs 2 printer using clear V4 resin, with a printing resolution of 0.1 mm. The SLA printing technique was chosen as it produces high-accuracy prints with a resolution of 0.05 mm. The material was chosen as it provides watertight objects and due to practical aspects of availability and previous experience for this printer. Printed objects were washed twice in isopropanol bath and, if needed, manually with a syringe. They were cured for 20 min in UV light at 60 ^◦^C. All phantoms were watertight tested for 48 h.

### Construction and characterization of printed grids

Characterization of the properties of the printed grids was performed using cubes and spheres. These were designed in Fusion 360 (Autodesk, Inc.) and exported to the printer as STL files. A base grid was designed in Rhinoceros 5.5.5 (McNeel).

To determine the mass density of the printing material, five cubes, each of 10 $\times$ 10 $\times$ 10 mm^3^, were printed with 100% infill material. After printing, their dimensions were determined with a calliper and their mass using a calibrated Toledo AC100 balance (Mettler Toledo). The mass density, $\rho ,$ was then calculated.

To investigate how the SPECT image signal was modulated when using grids with different spacing, five spheres with diameter 40 mm were designed. Spheres 1–4 contained grids, while sphere 5 was empty. A base grid was first designed, consisting of a cuboid of 10 $\times$ 10 $\times$ 10 cm^3^ with a wall thickness of 2 mm and a distance between walls of 2 mm. The shape of the base grid was then adjusted using a Boolean operation. A neck was added to the sphere to enable filling and a foot for stability at filling. To design grids with different infill volumes, the grid dimensions (wall thickness and spacing) were scaled linearly. The fillable-volume fraction, *FVF*, was defined according to

1 $$\begin{array}{*{20}c} {{\it{\text{FVF}}} = 1 - \frac{{V_{{{\text{grid}}}} }}{{V_{{{\text{no}} - {\text{grid}}}} }},} \\ \end{array}$$

where the volume of the container without grid, $V_{{{\text{no}} - {\text{grid}}}}$, was calculated from the design. The volume of each grid, $V_{{{\text{grid}}}}$, was determined both from the design ($V_{{{\text{grid}},{\text{d}}}} )$ and experimentally as $V_{{{\text{grid}},{\text{m}}}} = { }m_{{{\text{grid}}}} /\rho$, i.e., by weighing and using the determined mass density of the printing material. The grid mass $m_{{{\text{grid}}}}$ was determined as the mass difference between the respective empty container and the container with grid inserted. Spheres 1- 5 were designed with *FVF*s of 48%, 58%, 68%, 74% and 100% (i.e., empty).

Further characterization of the grids was performed using micro-CT imaging, to examine how the discrepancies between the designed and printed grids were distributed, and to verify weighing as a method to determine the real printed grid volume. Imaging was performed with 55 kV, 50 mAs, a filter of 100 ${\upmu }$m aluminium, a voxel size 0.05 $\times$ 0.05 $\times$ 0.05 mm^3^ and an acquisition time of 12 min. Each sphere was imaged separately.

#### SPECT imaging of spheres

SPECT imaging of spheres was performed using radionuclide stock solutions of ^99m^Tc-pertechnetate and [^177^Lu]Lu-DOTA-TATE. As the latter was obtained as remains from a patient therapy, it was mixed with 7 mg Na_4_EDTA to avoid free ^177^Lu sticking to the phantom walls. Activity measurements traceable to primary standard were taken using two activity meters: a Fidelis (Southern Scientific, Henfield, UK) and a cross-calibrated clinical activity meter (Capintec CRC15, Capintec Inc.) The mass of the inserted solution was determined using a traceably calibrated balance, as the weight difference between the empty and filled spheres. The activity concentration in the solution at the time of SPECT imaging, was 3.02 MBq/mL and 0.77 MBq/mL for ^99m^Tc and ^177^Lu, respectively. Spheres were mounted in a cylindrical Jaszczak phantom with diameter 21.6 cm and height 18.6 cm, using in-house made Perspex rods. For ^99m^Tc measurements, all five spheres were successfully prepared, while for ^177^Lu measurements, leakage prevented the use of sphere number 4 (*FVF* = 74%).

SPECT images were acquired with a GE discovery 670 SPECT/CT system. Projections were acquired in 128 $\times$ 128 matrices with a pixel size of 4.42 $\times$ 4.42 ${\text{mm}}^{2}$ in 60 angles over 360^◦^. For ^99m^Tc, low-energy high-resolution (LEHR) collimators were used, and a 15% energy window was centred at 140.5 keV with 45 s per projection. For ^177^Lu acquisitions, medium-energy general-purpose (MEGP) collimators were used, with a 15% energy window centred at 208 keV and 90 s per projection. CT acquisition was performed with 120 kVp and automatic exposure mAs.

SPECT reconstruction was performed using an OS-EM-based off-line reconstruction program, including scatter [23] and attenuation compensations, with resolution recovery (RR) using 16 iterations and 10 subsets, with voxel size 4.42 $\times$ 4.42 $\times$ 4.42 ${\text{mm}}^{3}$. Determination of the image calibration factor for ^177^Lu and ^99m^Tc was performed as previously described [24].

### Kidney- and thyroid-shaped phantoms

The grid technique was applied to two types of phantoms, representing kidneys and thyroids, both designed from two digital phantoms from the XCAT population [25, 26]. The kidney templates encompassed three compartments representing the renal pelvis, cortex and medulla. The pelvis was designed as solid material, and the cortex was void. For the renal medulla, consisting of separate pyramids, a grid was designed with an *FVF* chosen to yield a given mean activity concentration ratio with respect to the cortex. The targeted activity concentration ratio between medulla and cortex was 42% [19, 20].

**Fig. 1:**
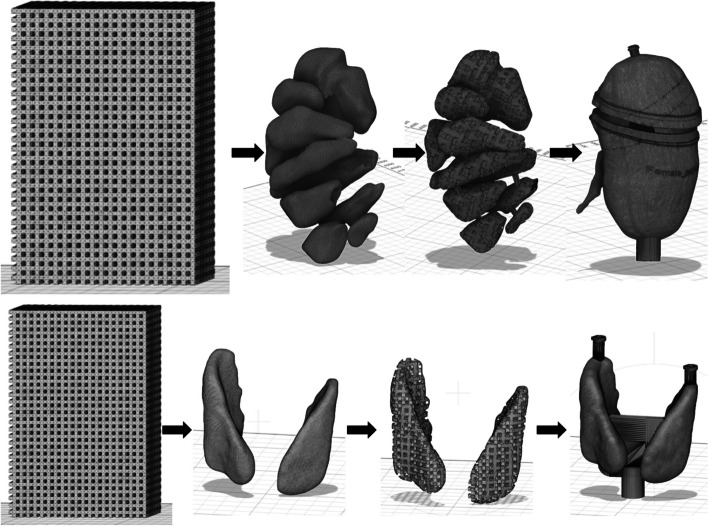
Schematic design steps for the construction of the grid-based phantoms. Upper row: kidney phantom with grid-based medulla structure, inserted into a separate cortex-shaped container with a filling hole on top and mounting neck on bottom. Bottom row: grid-based thyroid phantom with hot spots as voids (not shown), printed in an outer container with a plate to connect the two lobes, a filling hole on each lobe and a mounting neck at the bottom

Each kidney was printed as a container with an outer shape following the cortex, the pelvis placed at one side and a separate, insertable medulla part. To enable insertion of the medulla, the container was separated into two sections (Fig. 1), with a filling hole in the lid and a small cylinder for mounting at the bottom. The medulla pyramids were connected with small rods for stabilization. Technically, all parts were printed on the same building plate. After printing, the medulla section was weighed, and $V_{{{\text{grid}},{\text{m}}}} { }$ was calculated. Each kidney was assembled by mounting the medulla to the pelvis and gluing together the two sections of the outer container. The total volume of the outer phantom container (including pelvis) was 130 mL and 190 mL for the two kidney phantoms, respectively. Their fillable volumes, after insertion of the medulla grid and its supporting structures, were 116 mL and 167 mL, respectively.

The two thyroid phantoms were designed as two lobes with a connecting plate between them and a filling hole in each lobe (Fig. 1). Each lobe encompassed two compartments: an outer compartment composed of a grid and voids to mimic thyroid hot spots. The hot spots were designed from spherical shapes, with diameters between 15 mm and 20 mm. By guidance from clinical ^99m^Tc-pertechnetate thyroid images acquired for volume determination of the thyroid in treatments with Na[^131^I]I, spheres were manually positioned in the thyroid lobes of the XCAT phantom and then constrained and cut to fit into a given lobe. The grid was designed with an *FVF* chosen to yield a given mean activity concentration ratio between the thyroid and hot spot. The targeted activity concentration ratio was derived from clinical ^99m^Tc-pertechnetate thyroid images, and the *FVF* was determined as 50%.

Phantom 1 contained two hot spots in one lobe and one in the other, while phantom 2 contained one hot spot in one lobe and none in the other. Due to their small size, each thyroid phantom, including the internal grid, was printed as one connected entity. The grid volume, $V_{{{\text{grid}},{\text{m}}}}$, was determined following the same method as for the spheres. The outer phantom volumes were 19 mL and 22 mL, and the fillable volumes after insertion of the grid were 11.5 mL and 11.6 mL for the two thyroid phantoms, respectively.

#### SPECT imaging

The anthropomorphic phantoms were filled with radioactive solutions following the procedures described for spheres. The kidneys were filled with a ^177^Lu activity concentration of 0.2 MBq/mL at time of SPECT/CT, giving a total activity of 31.4 MBq and 21.7 MBq [20] for the two kidneys. The two kidneys were mounted in a Jaszczak phantom using an in-house manufactured Perspex plate and rods. The thyroids were filled with a ^99m^Tc activity concentration of 3.2 MBq/mL and 3.6 MBq/mL at time of SPECT, giving a total activity for the two lobes of 35.8 MBq and 40.0 MBq [27], for the two phantoms, respectively. The thyroids were mounted separately into a cylindrical in-house manufactured phantom with diameter 12 cm and height 16 cm, designed to mimic a patient neck.

Projections were acquired with settings as for the sphere SPECT acquisitions with ^177^Lu and ^99m^Tc for kidneys and thyroids, respectively. The time per projection was adjusted depending on the activity concentration in the objects. Thyroids were measured for 15 s per projection. For kidneys 120 projections were acquired with 180 s per projection, to achieve a signal-to-noise ratio in SPECT images that corresponded to typical activities and times per projection in patient imaging.

Tomographic images were reconstructed with settings as for sphere measurements, but both with and without the inclusion of RR. Reconstructions without RR were made with 4 iterations and 10 subsets, while when including RR 16 iterations and 10 subsets were used. Images of kidneys reconstructed without RR were filtered with a 3D Gaussian filter with a full width half maximum of 8.8 mm.

#### Monte Carlo simulations

To examine the ability of the grid-based phantoms to mimic non-uniform activity distributions in SPECT images, simulations were performed using the Simind Monte Carlo program [28]. The SPECT simulations were intended to match the physical phantom measurements and were made for two kidneys and two thyroids. The same XCAT computer phantoms as used for design of the 3D printed phantoms were used. For the kidneys, the activity concentrations assigned to the cortex and pelvis corresponded to those of phantom measurements. For the medulla, which for physical phantoms consisted of a grid, the assigned activity concentrations corresponded to the volume average over the medulla extension for the respective phantom. For the thyroids, the designed hot spots were included in the simulated geometry. Activity concentrations assigned to the hot spots corresponded to those used for measurement, while the activity concentrations for the remaining thyroid, consisting of a grid for the physical phantoms, corresponded to the average over their extension. Thus, the simulated geometries closely matched the physical phantom geometries, with only difference in how the non-uniform activity concentrations were created.

To facilitate the comparison of measured and simulated image data, the computer phantoms were first rotated and translated by guidance of CT images of the physical phantoms. The reoriented computer phantoms were placed in a voxelized cylinder of the same dimensions as used for physical phantom measurements. A uniform medium (water, 1.0 g/cm^3^) was used. The position of the cylinder in the image field of view was defined to approximately correspond to that of phantom measurements, and simulations were performed using the same projection distances as for phantom measurements. The simulated projections were scaled to correspond to the total activity and acquisition time of the measured projections, and Poisson-distributed noise was added using a pseudorandom number generator. SPECT images were reconstructed with the same settings as the measurements. The image calibration factors for ^177^Lu and ^99m^Tc for the simulated camera were determined following the same procedure as for the physical phantom measurements [24].

### Evaluation

The correspondence between the designed and printed grids was examined for spheres, kidney medulla inserts and thyroid phantoms. The relative deviation between the volume from design ($V_{{{\text{grid}},{\text{d}}}} )$ and experimental measurements ($V_{{{\text{grid}},{\text{m}}}} )$ was calculated according to

2 $$\begin{array}{*{20}c} {\Delta V = 1 - \frac{{V_{{{\text{grid}},{\text{d}}}} }}{{V_{{{\text{grid}},{\text{m}}}} }} } \\ \end{array}$$

where the volume determined by weighing was thus used as reference.

**Fig. 2:**
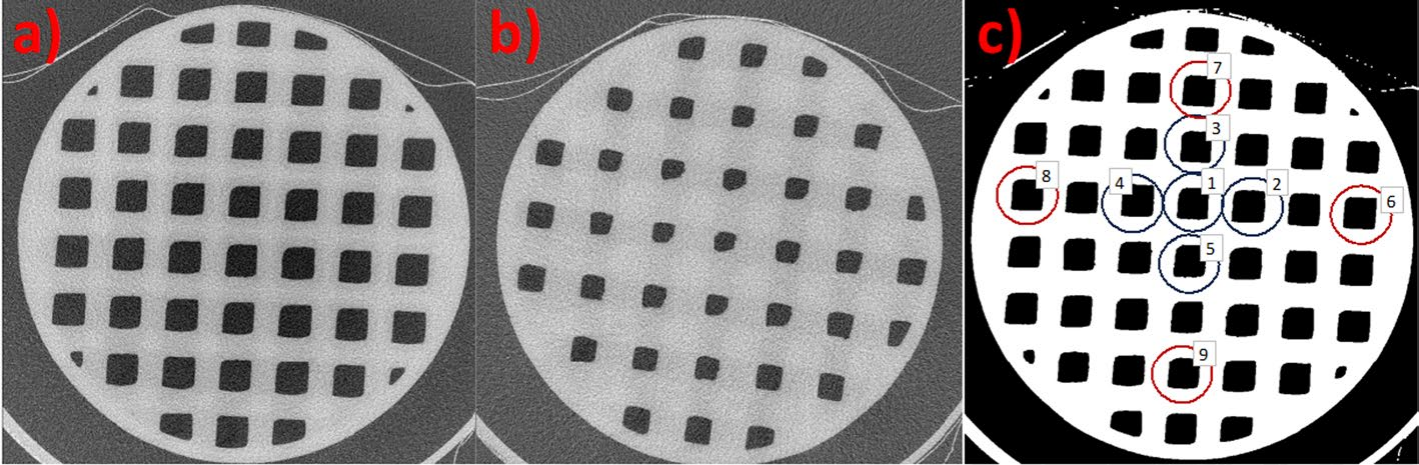
Example micro-CT images, acquired for grid characterization, of spheres with **a** high fillable-volume fraction (*FVF*) of *59%* and **b** low *FVF* of 27%. The printing inaccuracies with respect to the design were observed at the sharp edges of the grid crossings and were fractionally higher for the denser grids (low *FVF*). **c** Segmented mask of one sphere, including ROIs used to calculate the area ratio for central (ROIs 1–5) versus peripheral (ROIs 6–9) voids as a metric of the geometric variation of printing inaccuracies

The micro-CT images were visually inspected, to examine how possible printing inaccuracies were distributed over the grid (Fig. 2). To enable quantitative evaluation, micro-CT images were segmented. This was performed using a moment-preserving thresholding [29] followed by morphological opening and closing operations with 3 × 3 × 3 masks and exclusion of the sphere shell by adaption of an analytical sphere to the gradient of the resulting mask. The total volume of the printed grids was determined and the volumetric deviation determined using Eq. 2 with the volume from the micro-CT in place of $V_{{{\text{grid}},{\text{d}}}}$. The distribution of printing inaccuracies across grids was examined by calculating the areas of central and peripheral voids in a transversal slice at the centre of the spheres. A ratio of the central to peripheral areas was formed, giving a value that ideally should be unity. Five central and four peripheral regions of interest (ROIs) were defined (Fig. 2), and the areas were taken as the mean void areas across the ROIs representing the respective location.

The SPECT/CT images for the spherical inserts were used to examine practical aspects that could affect the filling of the grids with radioactive solution, for example formation of air bubbles which might affect the infill volume differently for the different grids. In the reconstructed SPECT/CT images, a spherical volume of interest (VOI) was delineated for each sphere, with volume according to the physical volume. The mean image signal in the VOI, corresponding to the sphere mean activity concentration, was determined, and the modulation contrast, $C_{{\text{M}}}$, was quantified according to:

3 $$\begin{array}{*{20}c} {C_{{\text{M}}} = \frac{{S_{{{\text{SP}},{\text{grid}}}} }}{{S_{{{\text{SP}},{\text{ no}} - {\text{grid}}}} }} } \\ \end{array}$$

where $S_{{{\text{SP}},{\text{grid}}}}$ and $S_{{{\text{SP}},{\text{no}} - {\text{grid}}}}$ are the respective mean image signals for spheres with a given *FVF* and for the sphere without a grid (*FVF* = 100%). The resolution-induced spill-out was assumed to be equal for spheres with and without grid, such that by taking the ratio this effect was cancelled. Thus, $C_{{\text{M}}}$ was regarded to mainly reflect the properties of the grid and its filling. The $C_{{\text{M}}}$ was plotted as a function of the *FVF*, and a linear relationship was determined using linear regression. Separate relationships were derived for ^99m^Tc and ^177^Lu.

Evaluation of the ability of the grid-based phantoms to provide realistic, non-uniform SPECT images, including blurring due to limited spatial resolution and ringing artefacts from the inclusion of RR in the reconstruction, was made by comparison of experimentally measured and Monte Carlo simulated images.

The kidney phantom from measurements and simulations was compared visually and quantitatively in three dimensions and by analysis of profiles in coronal slices. To mitigate effects of the remaining positional mismatch of measured and simulated phantoms, the profiles were calculated as the average activity concentration across the three central coronal slices. Quantitative comparison of measured and simulated images was made by determining the activity recovery, relevant for dosimetry in ^177^Lu PRRT. Recovery was defined according to

4 $$\begin{array}{*{20}c} {R = \frac{{A_{{{\text{SPECT}}}} }}{A}} \\ \end{array}$$

where $A_{{{\text{SPECT}}}}$ is the kidney activity derived from the SPECT images and $A$ is the activity from the phantom preparation or the setup of simulation. The VOIs were defined manually along the border of each kidney in the SPECT images. As the main purpose was to compare the measured and simulated images, inconsistencies due to VOI delineation were minimized by also including the renal pelvis in these VOIs.

For thyroid phantoms, comparison of measured and simulated ^99m^Tc SPECT images was made using maximum intensity projections (MIPs) to capture the overall image appearance of these small-sized objects. Quantitative comparison of measured and simulated images was made with respect to image-based volume quantification, relevant for the clinical use of thyroid ^99m^Tc-pertechnetate images. The sensitivity of the estimated volume was thus examined for different thresholds used for image segmentation, for measured and simulated SPECT images. For the purpose of comparison, the threshold was specified in terms of absolute activity concentration, instead of, e.g., a threshold relative to the maximum value. The threshold was varied in 100 equal steps of the maximum activity concentration in the image, and the volume of the enclosed region was calculated as the number of voxels multiplied by the voxel volume. As metric of agreement between curves from measured and simulated SPECT images, the Euclidean distance, $\overline{d}$, between corresponding curve sample points was calculated and the mean across all points determined. The threshold yielding the correct volume estimate was also calculated and expressed as a fraction of the maximum activity concentration in the SPECT images.

## Results

### Characterization of printed grids

From the solid printed cubes, the experimentally measured mass density of clear resin with after cure was obtained to (1.192 $\pm$ 0.009) g/cm^3^, in agreement with the density 1.185 g/cm^3^ provided by the manufacturer.

**Fig. 3:**
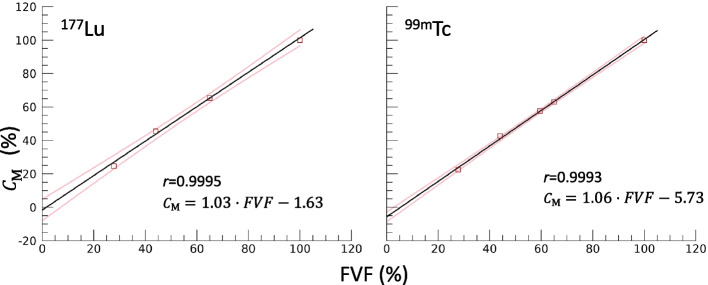
The modulation contrast, $C_{{\text{M}}}$ (Eq. 3), obtained from SPECT images of spheres for ^177^Lu (left) and ^99m^Tc (right), as function of the fillable-volume fraction, *FVF* (Eq. 1), determined by weighing ($V_{{{\text{grid}},{\text{m}}}}$). The fitted linear equations are indicated (black solid lines). The pink bands indicate one standard deviation around the fitted line

The grid volumes for spheres 1–4 determined by weighing, $V_{{{\text{grid}},{\text{m}}}}$, gave *FVF*s (Eq. (1)) of 28%, 44%, 60% and 65%, respectively. The maximum $\Delta V$ obtained was 28% (Eq. 2) for the sphere with *FVF* 28%. The $\Delta V$ between $V_{{{\text{grid}},{\text{m}}}}$ and micro-CT imaging (Fig. 2) were obtained to -3.1%, -4.5%, -6.2% and -8.1%, for increasing *FVF*s*.* By visual inspection of the micro-CT images, it was confirmed that inaccuracies with respect to the design were mainly associated with sharp edges and distributed across the grid geometry. The central-to-peripheral area ratios were obtained to 0.92, 0.95, 1.00 and 1.02 for increasing *FVF*s. Figure 3 shows results of the $C_{{\text{M}}}$ versus *FVF* data, obtained from the sphere SPECT/CT images for ^177^Lu and ^99m^Tc. The linear relationship had a slope near unity with coefficients with a narrow standard deviation (Fig. 3) and a Pearson’s correlation coefficient of *r* = 0.9995 and *r* = 0.9993 for ^177^Lu and ^99m^Tc, respectively. Additional file 1: Fig. S1 and S2 show SPECT images of the spheres and corresponding image profiles. Any patterns associated with the grid structures could not be identified in the SPECT images.

### Kidney- and thyroid-shaped phantoms

**Fig. 4:**
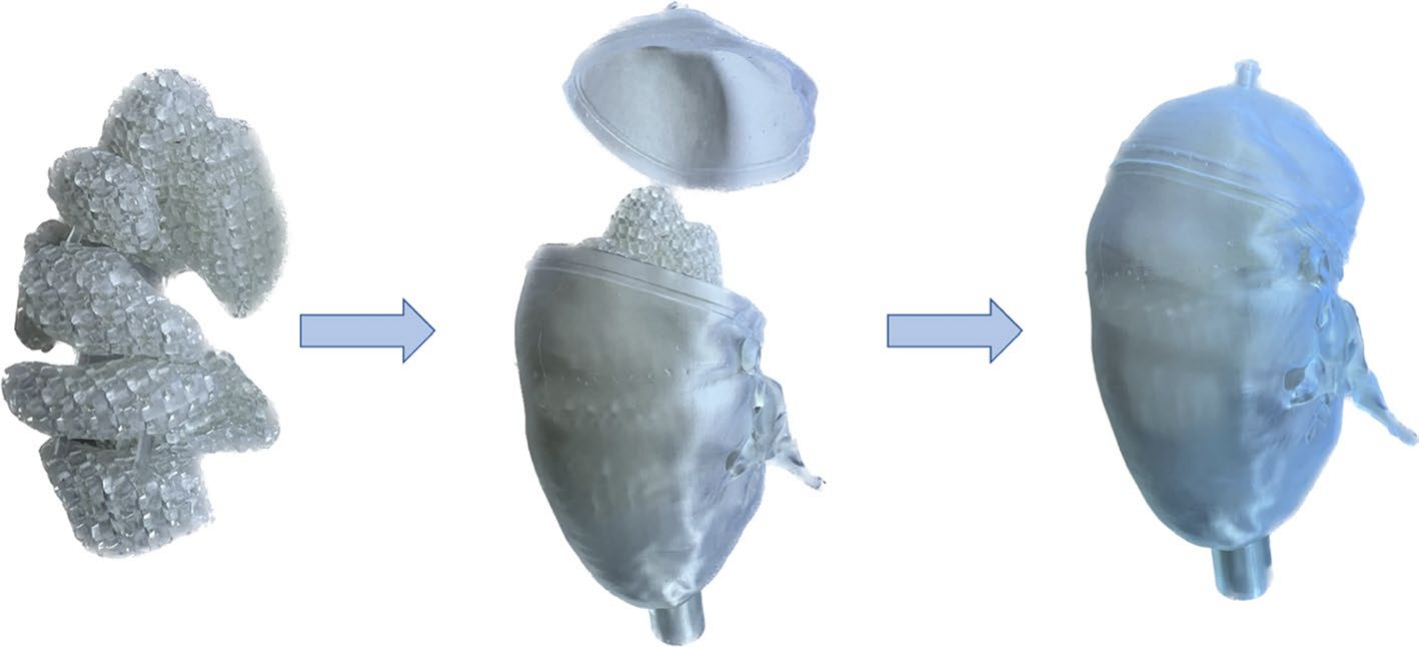
Photography of a 3D printed kidney phantom, including the grid-based medulla section that is mounted on the pelvis section and then inserted into the outer cortex-shaped container

**Fig. 5:**
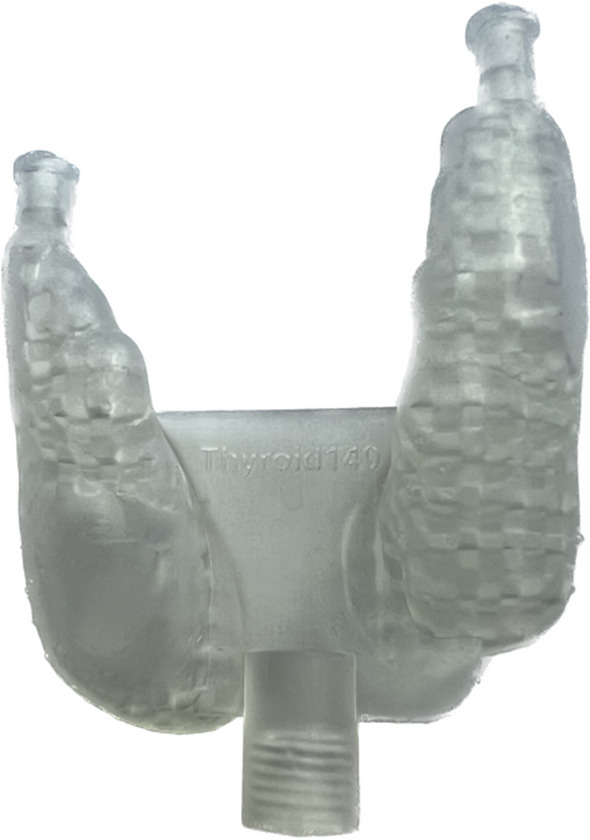
Photography of a 3D printed grid-based thyroid phantom, consisting of two lobes, a connecting plate between them and a filling hole for each lobe

**Fig. 6:**
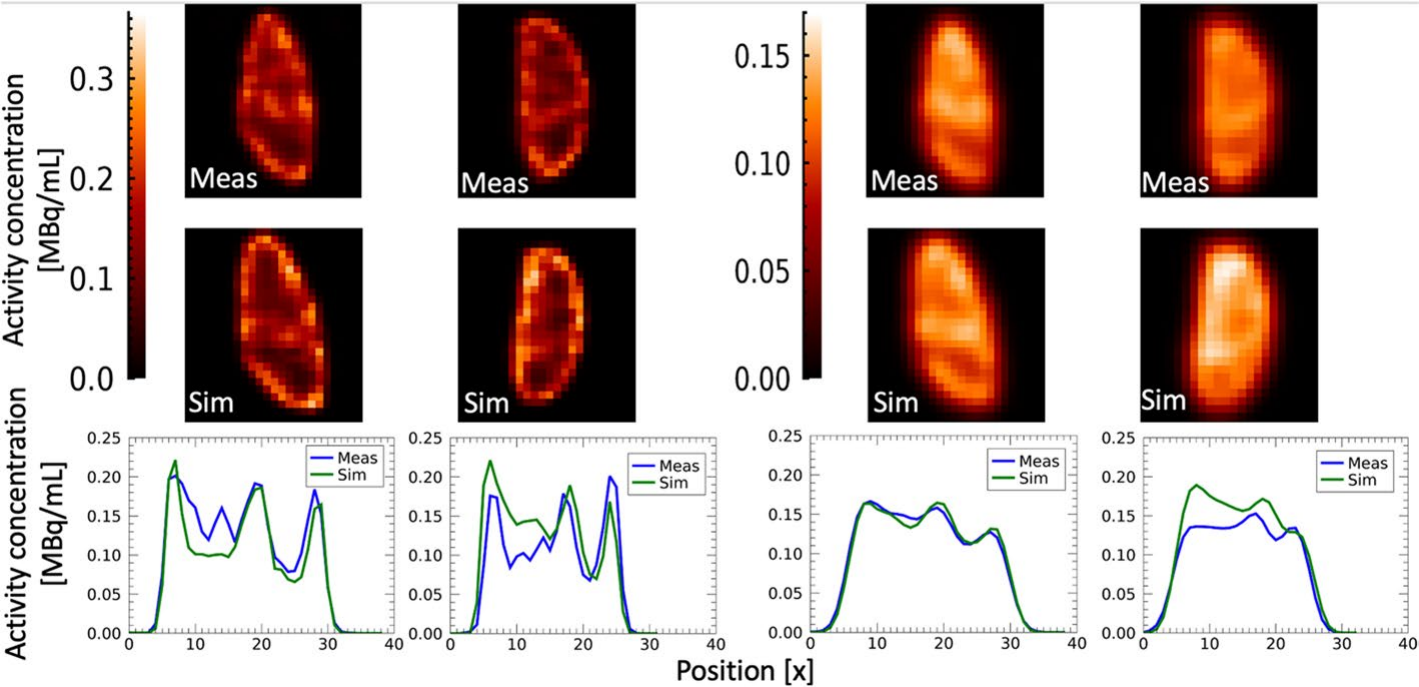
SPECT images obtained from measured (top row) and simulated (middle row) projections of one set of the kidney phantoms (left and right kidney). SPECT image reconstruction was made with (leftmost columns) and without (rightmost columns) resolution recovery, where the latter also included the application of a post-reconstruction Gaussian filter. Bottom row shows profiles for the measured (blue lines) and simulated (green lines) SPECT images, where each graph corresponds to the two images above, respectively

Figure 4 shows one of the printed kidneys. The total printing time was 14.5 h, and 100–150 mL resin material was required corresponding to a printing cost per kidney of approximately €25 (Formlabs Inc.). For the two medulla inserts, $\Delta V$ was obtained to 3% and 4% for the two phantoms, respectively. The *FVF*s determined by weighing ($V_{{{\text{grid}},{\text{m}}}} )$ were 36% and 38% (Fig. 5). Using the linear relationship for ^177^Lu, these *FVF*s corresponded to a $C_{{\text{M}}}$ between medulla and cortex of 36 $\pm$ 1% and 37 $\pm$ 1% for the two kidneys, respectively. Figure 6 shows central coronal ^177^Lu SPECT slices of the two kidneys with corresponding profiles, obtained from experimentally measured and Monte Carlo simulated image projection data. Additional comparisons of measured and simulated images are available in Additional file 1: Fig. S3–S7 where unfiltered images of reconstructions without RR are also included. Visual inspection and analyses of profiles confirmed that the images of the grid-based phantoms corresponded well to those obtained from Monte Carlo simulations, and enabled capturing of the non-uniform patterns as well as the limited image quality due to resolution blurring and ringing artefacts associated with RR. Table 1 shows quantitative results on the recovery obtained for the experimentally measured and simulated kidney SPECT images. For the same reconstruction settings, the recovery for the simulated and measured kidney phantoms agreed to within 4 percentage points.

**Table 1:** Recovery of the ^177^Lu activity in the kidney phantoms, for measured and Monte Carlo simulated projection data reconstructed with and without resolution recovery. VOIs were defined manually along the kidney border in SPECT images

Kidney phantom nr	Measured, including RR (%)	Simulated, including RR (%)	Measured, excluding RR (%)	Simulated, excluding RR (%)
1	90	90	76	75
2	91	87	77	73

**Fig. 7:**
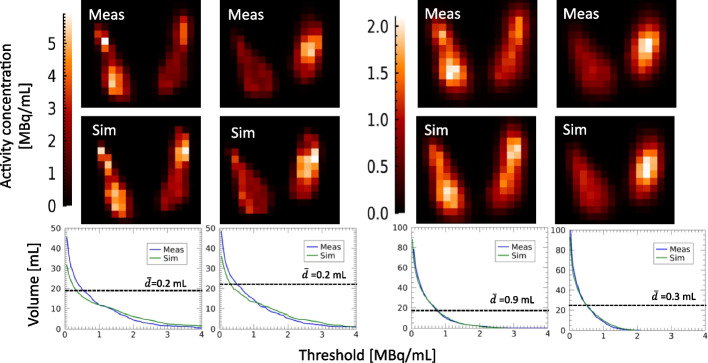
SPECT images obtained from measured (top row) and simulated (middle row) projections of the two thyroid phantoms, shown as maximum intensity projections. SPECT image reconstruction was made with (leftmost columns) and without (rightmost columns) resolution recovery. Bottom row shows the volume obtained by thresholding of the SPECT images as a function of the threshold value, expressed in terms of absolute activity concentration. Graphs include results from the measured (blue line), simulated (green line) SPECT images and the true phantom volume (horizontal dashed line), where each graph corresponds to the two images above, respectively. The mean of the Euclidean distance, $\overline{d}$, between the sample points on the curves are presented in the plots

Figure 5 shows one of the two thyroid phantoms. The total printing time for both thyroids was 7 h and required 20–25 mL resin material, corresponding to a cost per phantom of €3 (Formlabs Inc.). The $\Delta V$ was obtained to 13% and 15% for the two phantoms, respectively. The *FVF*s from $V_{{{\text{grid}},{\text{m}}}} { }$ were obtained to 61% and 53%. Using the linear relationship for ^99m^Tc, these corresponded to a $C_{{\text{M}}}$ between thyroid and hot spot of 59 $\pm$ 1% and 51 $\pm$ 1%, for the two phantoms, respectively. Figure 7 shows ^99m^Tc SPECT MIP images of the two thyroids, from measured and Monte Carlo simulated image projection data. When inspected in three-dimensional images, the measured images closely resembled those obtained from Monte Carlo simulations, when both including and excluding RR in the reconstruction. Figure 7 shows results of the thyroid volume estimated from SPECT images from measurements and Monte Carlo simulations, as a function of the threshold value used for thyroid delineation. Thresholds yielding a correct volume estimate are shown in Table 2. Generally, the results of the segmentation of measured and simulated data agreed well. For images reconstructed without RR, results were nearly identical, with subtle differences caused by noise. Slightly larger discrepancies were obtained when including RR in the reconstruction, likely associated with blurring and ringing artefacts. The mean Euclidian distance was obtained to 0.2 and 0.9 mL, for reconstructions with and without RR, respectively.

**Table 2:** Thresholds yielding a correct volume estimate for thyroid phantoms, expressed as a fraction of the maximum activity concentration in the thyroid SPECT image

Thyroid phantom nr	Measured, including RR (%)	Simulated, including RR (%)	Measured, excluding RR (%)	Simulated, excluding RR (%)
1	10	7	25	25
2	9	5	33	33

STL files of kidney and thyroid phantoms are available as Additional files 2, 3, 4, 5, 6, 7, 8 and 9.

## Discussion

A grid-based 3D printing technique was developed for the construction of anthropomorphic phantoms with non-uniform activity distributions, using only one stock solution for phantom filling. The grid-based technique was found to provide phantoms that effectively modulated the image contrast of SPECT images. By making Monte Carlo simulations in parallel with physical phantom measurements, it was verified that the image contrast could be modulated in a quantitatively correct manner. 

For the grid method to be useful for phantom construction, it was considered important to verify that there were no systematic artefacts or lumps resulting from 3D printing. In micro-CT images, deviations from the design were found to be mainly located at the sharp edges of the grid–wall crossings, uniformly across the grid structure. This thus provided a qualitative confirmation of the grid-based approach. Quantitatively, the *FVF*s obtained by analysis of micro-CT images and from weighing were generally good. In absolute terms, the volume deviations were comparably independent of the *FVF*, yielding a larger fractional deviation for the lowest grid volume (highest *FVF*). The central-to-peripheral area ratios from micro-CT images ranged from 0.92 to 1.02, where the lowest value was obtained for the lowest examined *FVF* (27%). Possibly this could indicate a non-uniformity in printing errors for this *FVF,* with smaller grid voids centrally than peripherally. However, based on these data an exact lower limit of the *FVF* could not be specified, as the obtained deviations would then also have to be set into relation to the uncertainties associated with image segmentation. The *FVF*s used for kidney and thyroid phantoms were 36%, 38% and 53%, 61%, respectively. In perspective of the obtained central-to-peripheral area ratios and the appearance of SPECT image profiles (Additional file 1: Fig. S1 and S2), any non-uniformity in printing errors for these *FVF*s was considered to have a negligible impact and weighing was considered verified as a method to determine the *FVF.*

The observed deviations between the design and printed grids introduced differences with respect to the *FVF*, such that a targeted *FVF* could not be exactly realized in practice. Larger deviations between the *FVF* from design and weighing were obtained for objects that were printed as one entity with the grid integrated (spheres and thyroids) than when the grid was printed separately (kidneys). This was a consequence of the different possibilities of cleaning the grid after printing, which was more difficult when it was enclosed in a container. To counteract these deviations, objects were oriented in a favourable angle for rinse of resin during print and were thoroughly cleaned from residual resin after print. Still, it was concluded that for situations where it was important that the *FVF* followed the design, the grid structure should preferably be printed separately. The downside of this approach was, however, that the outer phantom container had to be printed in two parts and assembled after grid insertion, which put demands on the mounting precision and the assurance of watertightness of the phantom. Similar deviations from the design have been observed when using STL printers, for example by Gillet et al. [3] who measured diametral deviations of 3D printed spheres, and reported a maximum deviation of 4%. 

An additional aspect to consider was the risk for trapped air bubbles in the grid, in view of the small cavities and large area with respect to surface tension. This could potentially affect the $C_{{\text{M}}}$ and introduce nonlinearity in the relationship between the *FVF* and $C_{{\text{M}}}$. Practical measures taken to reduce this risk was to colour the radioactive solution to enable visual inspection and to thoroughly shake the phantoms at filling. The results of the sphere measurements for ^177^Lu and ^99m^Tc confirmed that the relationships between the *FVF* and $C_{{\text{M}}}$ were linear. Thus, together these characterizing experiments showed that i) printing deviations were evenly distributed across the grid, ii) the *FVF* could thus be determined by weighing, and iii) the *FVF* was linearly related to the $C_{{\text{M}}}$ over a relevant range of volumes and activity concentrations. 

The capability of the printed objects to mimic anthropomorphic structures and act as relevant test geometries for quantitative SPECT methods was examined by comparison with images obtained from Monte Carlo simulation. These simulated images were based on similar geometries and activity distributions as the printed objects, with the only difference in how the regions of non-uniform activity were generated, using a grid for printed phantoms and a compartmentalized geometry for simulated images. This approach thus allowed for both qualitative and quantitative verification of the SPECT images for the printed objects. For both thyroid phantoms filled with ^99m^Tc and kidney phantoms filled with ^177^Lu, the activity distributions in the measured and simulated SPECT images corresponded well, in terms of both the quantitative values and the overall patterns. 

For kidney images (Fig. 6), profiles could not be exactly matched between simulated and measured images, but the trends, including the degree of blurring, were similar for both images reconstructed with and without RR. Small differences were observed at the top of the kidneys, which were caused by some minor adjustments made to enable mounting of the medulla grid into the outer cortex structure of the 3D printed phantoms. Furthermore, small misplacements of the medulla grid may have occurred due to imprecision in this mounting. The recoveries obtained for measured and Monte Carlo simulated projection data (Table 1) agreed to within 4 percentage points, which, given that image delineation was performed manually, was considered good. However, this could be compared to recoveries calculated for standard geometry phantoms (spheres) of the same volume (130 mL and 190 mL) for the same two variations of reconstruction settings [30, 31]. This gave recoveries for a 130 mL sphere of 0.91 and 0.82 and for a 190 mL sphere 0.93 and 0.85 reconstructed with and without RR, respectively. This corresponds to a maximum deviation in recovery coefficient between simulated non-uniform anthropomorphic kidney phantom 1 and standard geometry sphere of 19%. A similar comparison was made by Tran-Gia et al. [12] where a maximum deviation of 31.7% was observed for their two-compartment non-uniform kidney phantom and standard geometry sphere. This large deviation was not reproduced using our phantoms. 

For the thyroid images (Fig. 7), the quantitative comparison was made in terms of the volume resulting from thresholding for various choices of thresholds, corresponding to the clinical use of these images. Generally, results agreed well between simulated and experimentally measured images. For images reconstructed with RR, small local deviations were obtained as a result of the modified noise texture and enhanced local contrast introduced by RR, which combined with thresholding produced slightly variable results (Table 2, Fig. 7). The pronounced sensitivity of the estimated volume to the choice of threshold can be noted.

Apart from geometry differences between printed and simulated phantoms, a factor that was expected to affect this comparison was the use of resin for the printed phantoms, for which the density is slightly higher than the water density used for simulation. However, effects of these slightly different attenuation conditions could not be identified in the images, and it was concluded that the attenuation correction managed to cover this aspect. An alternative to the resin used herein could be to use an FDM printer and a printing material with attenuation properties more similar to that of water. However, such a strategy would require some post-printing processing, e.g., using glue or silicone, of the object to ensure a watertight phantom. 

Cost and flexibility are the main advantages when comparing 3D printed anthropomorphic phantoms to commercially available phantoms. The printed kidney and thyroid phantoms did all four together cost less than €100 in material. However, one might consider other costs associated with 3D printed phantoms beyond the price of the printing material. The different components for printing contribute to the total cost for a project such as this, e.g., printer, washer and cure, which together sum up to around €5000 (Formlabs Inc.). This equipment, i.e., printer, cleaning tools and machine for curing, is more expensive than for example equipment for FDM printing techniques, but provides a higher printing accuracy which was required for printing this grid structure and a more sophisticated process chain without glue. For this project, a collaboration with a 3D printing centre was developed which avoided the expensive and time-consuming start-up process. Such a collaboration gave access to machines, laboratories, materials and expertise to a cost of approximately €2500 per year. 

The grid-based technique provided phantoms of realistic shapes and activity distributions. These phantoms will be used for evaluation of image-based methods for quantification of activity or volume, for patient-specific dosimetry in radionuclide therapy. Method evaluation based on representative geometries is considered important to support comparison and pooling of patient data from different centres, for example in multi-centre studies.

## Conclusion

It is feasible to use 3D printed grid-based phantom structures to modulate the contrast of SPECT images, using a single stock solution for phantom preparation. The modulation contrast has been found to be linearly related to the filling volume fraction of the grids, and by comparison with Monte Carlo simulated SPECT images, the produced phantom geometries have been qualitatively and quantitatively verified. These phantoms open for experimental evaluation of methods for quantitative SPECT in geometries with non-uniform activity distributions.

### Supplementary Information

**Additional file 1.** Supplement to 3D printed non-uniform anthropomorphic phantoms for quantitative SPECT.

**Additional file 2.** kidney1_lid.

**Additional file 3.** kidney1_bottom.

**Additional file 4.** kidney1_medulla_grid.

**Additional file 5.** kidney2_lid.

**Additional file 6.** kidney2_bottom.

**Additional file 7.** kidney2_medulla_grid.

**Additional file 8.** thyroid1.

**Additional file 9.** thyroid2.

## Data Availability

All data generated or analysed during this study are included in this published article and its supplementary information files.

## Abbreviations

$$C_{{\text{M}}}$$: Modulation contrast
CT: Computed tomography
FDM: Fused deposition modelling
MRI: Magnetic resonance imaging
PET: Positron emission tomography
PVE: Partial volume effect
RR: Resolution recovery
SLA: Stereolithography
SPECT: Single photon emission computed tomography
STL: Standard tessellation language
FVF: Fillable-volume fraction
VOI: Volume of interest
ROI: Region of interest

## References

[1] El-Ali H, Ljungberg M, Strand S-E, Palmer J, Malmgren L, Nilsson J (2003). Calibration of a radioactive ink-based stack phantom and its applications in nuclear medicine. Cancer Biother Radiopharm.

[2] De Schepper S, Gnanasegaran G, Dickson JC, Van den Wyngaert T (2021). Absolute quantification in diagnostic SPECT/CT: the phantom premise. Diagnostics.

[3] Gillett D, Marsden D, Ballout S, Attili B, Bird N, Heard S (2021). 3D printing 18F radioactive phantoms for PET imaging. EJNMMI Phys.

[4] Läppchen T, Meier LP, Fürstner M, Prenosil GA, Krause T, Rominger A (2020). 3D printing of radioactive phantoms for nuclear medicine imaging. EJNMMI Phys.

[5] Gear JI, Cummings C, Sullivan J, Cooper-Rayner N, Downs P, Murray I (2020). Radioactive 3D printing for the production of molecular imaging phantoms. Phys Med Biol.

[6] Tran-Gia J, Denis-Bacelar AM, Ferreira KM, Robinson AP, Calvert N, Fenwick AJ (2021). A multicentre and multi-national evaluation of the accuracy of quantitative Lu-177 SPECT/CT imaging performed within the MRTDosimetry project. EJNMMI Phys.

[7] Woliner-van der Weg W, Deden LN, Meeuwis AP, Koenrades M, Peeters LH, Kuipers H (2016). A 3D-printed anatomical pancreas and kidney phantom for optimizing SPECT/CT reconstruction settings in beta cell imaging using 111 In-exendin. EJNMMI Phys.

[8] Price E, Robinson AP, Cullen DM, Tipping J, Calvert N, Hamilton D (2019). Improving molecular radiotherapy dosimetry using anthropomorphic calibration. Physica Med.

[9] Sgouros G, Bolch WE, Chiti A, Dewaraja YK, Emfietzoglou D, Hobbs RF (2021). ICRU report 96, dosimetry-guided radiopharmaceutical therapy. J ICRU.

[10] Liow J-S, Strother S (1993). The convergence of object dependent resolution in maximum likelihood based tomographic image reconstruction. Phys Med Biol.

[11] Pfaehler E, van Sluis J, Merema BB, van Ooijen P, Berendsen RC, van Velden FH (2020). Experimental multicenter and multivendor evaluation of the performance of PET radiomic features using 3-dimensionally printed phantom inserts. J Nucl Med.

[12] Tran-Gia J, Lassmann M (2018). Optimizing image quantification for 177Lu SPECT/CT based on a 3D printed 2-compartment kidney phantom. J Nucl Med.

[13] Maidment A, Christofides S, Dance D, Ng K, Kesner A, McLean D. A new IAEA handbook for teachers and students: Diagnostic radiology physics. 2010.

[14] Cerviño L, Soultan D, Cornell M, Yock A, Pettersson N, Song WY (2017). A novel 3D-printed phantom insert for 4D PET/CT imaging and simultaneous integrated boost radiotherapy. Med Phys.

[15] Theisen A-L, Lassmann M, Tran-Gia J (2022). Toward a patient-specific traceable quantification of SPECT/CT-based radiopharmaceutical distributions. J Nucl Med.

[16] Ljungberg M, Celler A, Konijnenberg MW, Eckerman KF, Dewaraja YK, Sjögreen-Gleisner K (2016). MIRD pamphlet no. 26: joint EANM/MIRD guidelines for quantitative 177Lu SPECT applied for dosimetry of radiopharmaceutical therapy. J Nucl Med.

[17] Dewaraja YK, Frey EC, Sgouros G, Brill AB, Roberson P, Zanzonico PB (2012). MIRD pamphlet no. 23: quantitative SPECT for patient-specific 3-dimensional dosimetry in internal radionuclide therapy. J Nucl Med.

[18] Dewaraja YK, Ljungberg M, Green AJ, Zanzonico PB, Frey EC, Bolch WE (2013). MIRD pamphlet no. 24: guidelines for quantitative 131I SPECT in dosimetry applications. J Nucl Med.

[19] De Jong M, Valkema R, Van Gameren A, Van Boven H, Bex A, Van De Weyer EP (2004). Inhomogeneous localization of radioactivity in the human kidney after injection of [111In-DTPA] octreotide. J Nucl Med.

[20] Brolin G, Gustafsson J, Ljungberg M, Gleisner KS (2015). Pharmacokinetic digital phantoms for accuracy assessment of image-based dosimetry in 177Lu-DOTATATE peptide receptor radionuclide therapy. Phys Med Biol.

[21] Stokkel MP, Handkiewicz Junak D, Lassmann M, Dietlein M, Luster M (2010). EANM procedure guidelines for therapy of benign thyroid disease. Eur J Nucl Med Mol Imaging.

[22] Sjögreen Gleisner K, Chouin N, Gabina PM, Cicone F, Gnesin S, Stokke C (2022). EANM dosimetry committee recommendations for dosimetry of 177Lu-labelled somatostatin-receptor-and PSMA-targeting ligands. Eur J Nucl Med Mol Imaging.

[23] Frey EC, Tsui B, editors. A new method for modeling the spatially-variant, object-dependent scatter response function in SPECT. 1996 IEEE Nuclear Science Symposium Conference Record; 1996: IEEE.

[24] Ljungberg M, Gleisner KS (2018). 3-D image-based dosimetry in radionuclide therapy. IEEE Trans Radiat Plasma Med Sci.

[25] Segars WP, Sturgeon G, Mendonca S, Grimes J, Tsui BM (2010). 4D XCAT phantom for multimodality imaging research. Med Phys.

[26] Segars W, Bond J, Frush J, Hon S, Eckersley C, Williams CH (2013). Population of anatomically variable 4D XCAT adult phantoms for imaging research and optimization. Med Phys.

[27] Al-Muqbel KM (2022). Utility of 99mtechnetium pertechnetate thyroid scan and uptake in thyrotoxic patients: jordanian experience. World J Nucl Med.

[28] Ljungberg M, Strand S, King M (1998). The SIMIND Monte Carlo program: Monte Carlo calculation in nuclear medicine: Applications in diagnostic imaging Bristol.

[29] Tsai W-H (1985). Moment-preserving thresolding: a new approach. Comput Vis Graphics Image Process.

[30] Roth D, Gustafsson J, Warfvinge CF, Sundlöv A, Åkesson A, Tennvall J (2022). Dosimetric quantities in neuroendocrine tumors over treatment cycles with 177Lu-DOTATATE. J Nucl Med.

[31] Gustafsson J, Sundlöv A, Sjögreen GK (2017). SPECT image segmentation for estimation of tumour volume and activity concentration in 177 Lu-DOTATATE radionuclide therapy. EJNMMI Res.

