# Supplementary material for: 3D printed non-uniform anthropomorphic phantoms for quantitative SPECT
Source: EJNMMI Phys. 2024 Jan 22;11:8. doi: 10.1186/s40658-024-00613-7 (PMC10803701; doi:10.1186/s40658-024-00613-7)
Supplement: Supplementary file 1 — Additional file 1. Supplement to 3D printed non-uniform anthropomorphic phantoms for quantitative SPECT. [file 40658_2024_613_MOESM1_ESM.docx]

Supplement to 3D printed non-uniform anthropomorphic phantoms for quantitative SPECT

Lovisa Jessen^1*^, Johan Gustafsson^1^, Selma Curkic Kapidzic ^1,2^, Michael Ljungberg ^1^, Muris Imsirovic ^2^, Katarina Sjögreen Gleisner ^1^

^1^Medical radiation Physics, Lund, Lund University, Lund, Sweden, ^2^Radiation Physics, Department of Hematology, Oncology and Radiation Physics, Skåne University Hospital, Sweden.

*Corresponding author:

Lovisa Jessen

[Lovisa.jessen@med.lu.se](mailto:Lovisa.jessen@med.lu.se)

Figure S1 shows SPECT images of spheres with grids. The image appearance was examined from profiles placed centrally in the spheres as indicated in Figure S1. No patterns from the grid structures were observed in the profiles (Figure S2).

**Figure S1**. Coronal SPECT slices for spheres with grids with different dimensions (see text). The yellow line indicates the profile position. A) Five spheres filled with ^99m^Tc, b) Four spheres filled with ^177^Lu.

**Figure S2**. Profiles of spheres imaged with ^99m^Tc (left) and ^177^Lu (right).

Figure S3 shows images reconstructed without RR without filtering. As noted, the noise level was too high to draw any conclusions about the activity distribution. For this reason, these images were post-filtered with a gaussian filter of FWHM=8.8 mm.

**Figure S3**. Transversal and coronal slices of ^177^Lu SPECT of kidney phantoms reconstructed without resolution recovery and without any filtering: a) coronal slice, measured b) transversal, measured, c) coronal, simulated, d) transversal, simulated.

**Figure S4**. Transversal slices 56, 57, 58 (from left) of experimentally measured ^177^Lu SPECT of the left kidney phantom, reconstructed with resolution recovery (upper row) and without resolution recovery and a gaussian filter of FWHM=8.8 mm (lower row).

**Figure S5**. Transversal slice 51, 52, 53 (from left) of experimentally measured ^177^Lu SPECT of the left kidney phantom. Reconstructed with resolution recovery (upper row) and reconstructed without resolution recovery and gaussian filter of FWHM=8.8 mm (lower row).

**Figure S6.** Transversal slice 60, 61, 62 (from left) of Monte Carlo simulated ^177^Lu SPECT of the left kidney phantom. Reconstructed with resolution recovery (upper row) and without resolution recovery with gaussian filter of FHWM=8.8 mm (lower row).


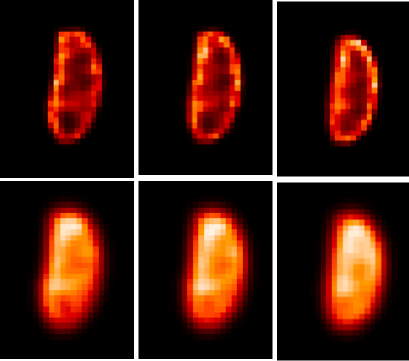


**Figure S7**. Transversal slice 56, 57, 58 (from left) of Monte Carlo simulated ^177^Lu SPECT of the left kidney phantom. Reconstructed with resolution recovery (upper row) and without resolution recovery with gaussian filter FWHM=8.8 mm (lower row).
